# Physiological benefits of lung recruitment in the semi-lateral position after laparoscopic surgery: a randomized controlled study

**DOI:** 10.1038/s41598-022-04841-8

**Published:** 2022-03-10

**Authors:** Eun Jung Oh, Eun Ji Lee, Burn-young Heo, Jin Huh, Jeong-Jin Min

**Affiliations:** 1 Department of Anesthesiology and Pain Medicine, Samsung Medical Center, Sungkyunkwan University School of Medicine, Irwon-ro 81, Gangnam-gu, Seoul, 06351 Korea; 2 Department of Anesthesiology and Pain Medicine, Seongnam Citizens Medical Center, Seongnam, 13290 Korea; 3 Department of Anesthesiology and Pain Medicine, Kangwon National University Hospital, Chuncheon, 24341 Korea

**Keywords:** Medical research, Clinical trial design, Randomized controlled trials

## Abstract

We evaluated the physiological benefits of performing lung recruitment maneuver (LRM) in the semi-lateral position compared in the supine position. Seventy-nine patients undergoing laparoscopic prostatectomy were randomly assigned to either the supine or semi-lateral group according to body position during the LRM. At the end of surgery, LRM (35 cmH_2_O for 20 s) was performed twice in the assigned posture. The primary outcome was the maximal decrease in systolic arterial pressure during LRM. Secondary outcomes were changes in PaO_2_/FiO_2_ and the regional lung volume distribution after LRM. The decrease in systolic arterial pressure during the LRM was significantly higher in the supine group than in the semi-lateral group (mean ± standard deviation, [−] 27.6 ± 14.6% vs. [−] 18.6 ± 9.9%, *P* = 0.001). Improvement in PaO_2_/FiO_2_ ratio after the LRM was evident in both groups but was more prominent in the semi-lateral group than in the supine group (median [interquartile range], 39.3% [20.2, 63.6] vs. 18.2% [8.4, 29.2], *P* = 0.001). Among the horizontal lung divisions, regional lung volume in the most dependent portion (the dorsal division) was significantly increased after the LRM only in the semi-lateral group (*P* = 0.024). Performing lung recruitment in a semi-lateral position protected against hemodynamic deterioration during the LRM and increased regional lung ventilation in the dependent portion of the lung, leading to an improvement in arterial oxygenation after laparoscopic procedures.

**Trial registration** Clinical Research Information Service (https://cris.nih.go.kr/). Identifier: KCT0003756.

## Introduction

Atelectasis occurs in most patients undergoing general anesthesia, and this is more prominent in patients undergoing laparoscopic surgery^1,2^. In laparoscopic surgery, abdominal insufflation combined with the Trendelenburg position exacerbates compressive atelectasis, which are known to be dominant in the dependent portion of the lung. These changes may theoretically lead to intrapulmonary shunting and impaired gas exchange, increasing the risk of post-operative pulmonary complications (PPCs).

Lung recruitment maneuvers (LRMs) are useful in re-opening the atelectatic lung area^3^ and has been found to effectively reduce PPCs^4,5^. However, the LRM is a complex process whose effects depend on the amount of recruitment achieved without over-distention. The high recruitment airway pressures to achieve the maximal recruitment possibly induces over-distension in the non-atelectatic area, which is usually the non-dependent portion of the lung^6^. Therefore, LRMs thought to be effective are sometimes accompanied by transient hemodynamic instability^7–9^ and collapsed capillaries in the over-distended alveolar region, increasing the shunt of pulmonary blood flow^10,11^. Therefore, more effective LRM strategies are needed to re-aerate the most atelectatic lung regions without over-distension of the non-atelectatic lung while minimizing hemodynamic deterioration.

Previous studies have introduced LRM in the prone position among ARDS patients^6,12,13^, and changing the body position from supine to lateral during the LRM in anesthetized pediatric patients which effectively re-opened the collapsed lung area at lower airway pressures^14^. Although there have been studies evaluating LRM in different positions, to the best of our knowledge, so far, no study has demonstrated it among adult patients in the operating room. Therefore, we hypothesized that performing the LRM in a semi-lateral position would have hemodynamic benefits and improve alveolar recruitment in the most dependent region compared with the supine position. This position is easier to apply than the prone or full lateral position and reduces surface contact between the patient’s torso and the operating table, which may reflect less pressure on the heart during lung inflation.

In this randomized-controlled study, we compared the physiological effects of the LRM in semi-lateral position with the effects of LRM in supine position among patients undergoing robot-assisted laparoscopic radical prostatectomy.

## Methods and materials

The study protocol was approved by the Institutional Review Board of Samsung Medical Center on 27 March 2019 (SMC-2018-12-087-001) and was registered on 12 April 2019 at the Clinical Research Information Service (https://cris.nih.go.kr/; KCT0003756). Written informed consent was provided by all participants and all methods were performed in accordance with the relevant guidelines and regulations.

### Patients and randomization

Between April 2019 and January 2020 in a tertiary hospital, adult patients undergoing robot-assisted laparoscopic radical prostatectomy (RALRP), with a body mass index (BMI) < 35 kg m^−2^ and an ASA physical status of I to III, were enrolled. Patients with pre-operative lung disease; severe abnormal pulmonary function tests; history of previous lung surgery or requiring continuous infusion of a cardiovascular drug; high intracranial pressure; and inability to undergo electrical impedance tomography (EIT) were excluded. Eligible patients were randomized 1:1 to either the supine group (group S) or the semi-lateral group (group L) using computer-generated numbers by a statistician not involved in patient screening or enrolment.

### Anesthesia and emergence

Anesthetic management was standardized except for patient posture during the LRM. After initiating standard monitoring, anesthesia was induced with intravenous thiopental sodium (5 mg kg^−1^). Neuromuscular blockade was achieved using rocuronium (0.6–1.0 mg kg^−1^) at anesthesia induction and a moderate block was maintained throughout the study period. Following intubation, mechanical ventilation with a tidal volume of 8 ml per predicted body weight (kg), 50% inspiratory oxygen fraction (FiO_2_), and positive end-expiratory pressure (PEEP) of 5 cmH_2_O were provided (Ventilator: Carestation 650, Datex-Ohmeda Inc., WI, USA). Arterial blood pressure was monitored and cardiac index (CI), and stroke volume variation (SVV) were measured using an EV1000™ (Edwards Lifesciences LLC, CA, USA). The crystalloid infusion during surgery was adjusted to maintain normovolemic status, with a pulse pressure variation (PPV) and SVV less than 15%.

During robotic procedure, patients were placed in the lithotomy position with extreme Trendelenburg (approximately 25°–30°) and peritoneal insufflation was achieved with 13–15 mmHg of intra-abdominal carbon dioxide. Mechanical ventilation was changed to pressure-controlled mode, and airway pressure was adjusted to a tidal volume of 8 ml per predicted body weight (kg). A PEEP of 5 cmH_2_O was maintained for the duration of peritoneal insufflation. At the end of surgery, the LRM was performed in the allocated position before the emergence from anesthesia.

### Intervention

Before the LRM, volume status and hemodynamic stability were evaluated using CI, SVV, and PPV, while the mean arterial pressure was required to be higher than 60 mmHg, with a heart rate greater than 60 beats per minute (bpm)^7^. Also, patients who received continuous cardiovascular drugs were eliminated before the intervention.

**Figure 1:**
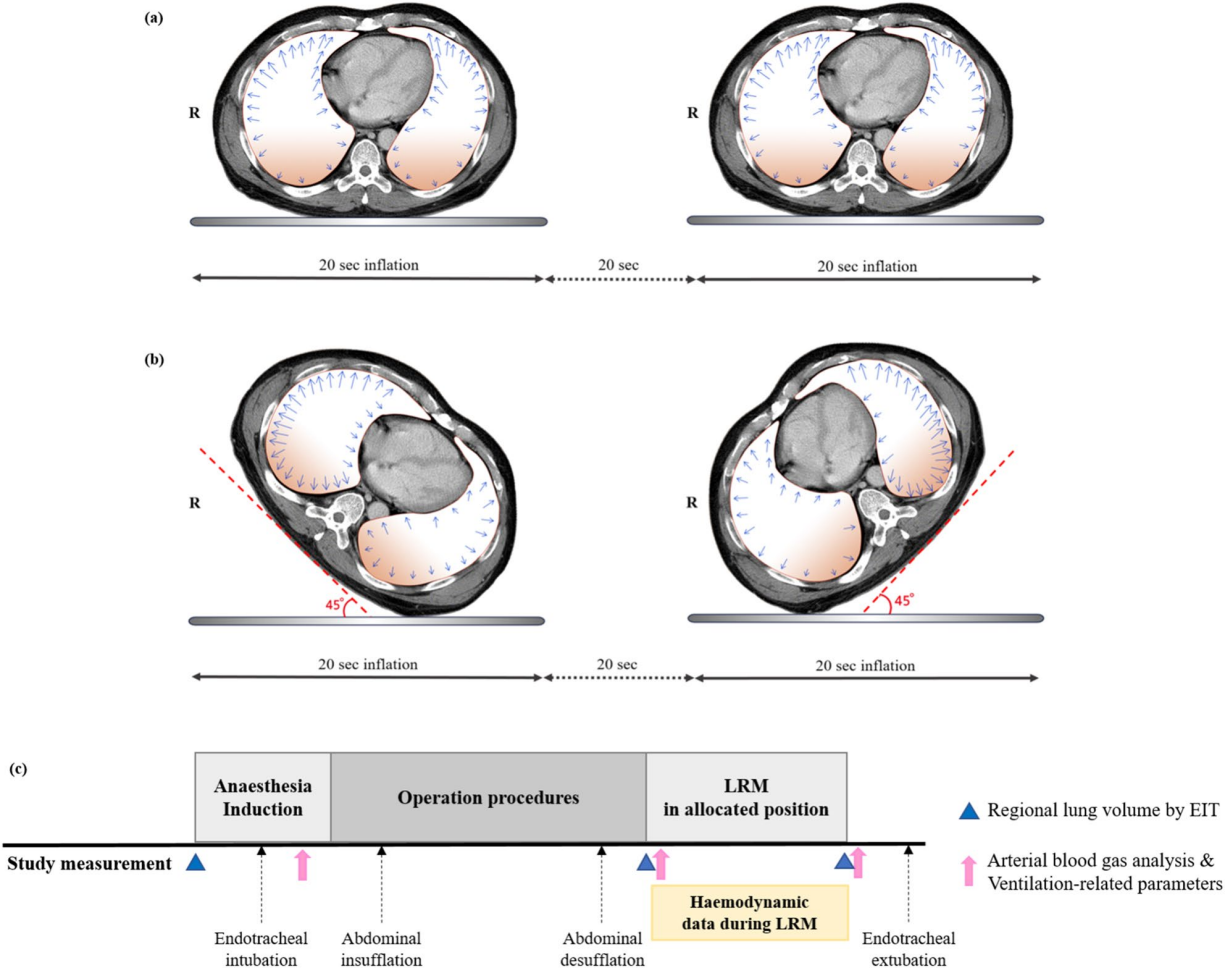
Lung recruitment maneuver in (**a**) supine position and (**b**) semi-lateral position (**L**eft lateral decubitus position and right lateral decubitus position). The dark color gradation inside the lung refers to the dependent portion of the lung during surgery, where atelectasis often occurs. In addition, the blue arrows refer to the hypothetical regional distribution of lung volume during lung recruitment maneuver. (**c**) Study protocol.

The LRM was performed with sustained inspiratory pressure at 35 cmH_2_O for 20 s after skin closure at the end of surgery by one researcher (EJ Lee) (Fig. 1). In the supine group (group S), the LRM was conducted in the supine position twice, with a 20-s pause between the maneuvers. In the semi-lateral group (group L), the patient was lifted into the semi-lateral position at an angle of 45° above the surgical table by assistant researchers who participated in the surgery. A thin surgical bed sheet laid underneath the patient throughout the surgery was used to wrap the patient’s body and maintain the patient’s body straight during positioning. By lifting the body wrapped with the surgical sheet, the patient’s limbs moved simultaneously with the body. Just a few more seconds were required to prepare for this patient positioning. The LRM was first conducted at left and then at right semi-lateral position, with a 20-s pause between the LRMs. During semi-lateral position the correct placement of the EIT belt was controlled by the surgical bed sheet. The regional lung volume distribution was collected in the supine position immediately after the LRM and PEEP was applied after the data acquisition. All LRMs were conducted with a 50% inspiratory oxygen fraction_._

### Study variables and data acquisition

To measure LRM-related changes in regional lung volume distribution and end-expiratory lung impedance (EELI), we used EIT (Dräger PulmoVista^®^ 500, Lubecca, Germany). EIT is a non-invasive, real-time imaging method used to monitor functional lung changes by measuring changes in thoracic impedance through a 16-electrode belt applied around the fourth to sixth intercostal spaces. Previously, the regional distribution of lung ventilation measured via EIT showed a linear correlation with lung volume measured via Single-photon emission computed tomography (SPECT)^6,15^. In addition, EELI reflects the functional residual capacity (FRC), which is affected by atelectasis^16^. Therefore, in the current study, changes in EELI defined as the difference in EELI before and after the LRM, and the distribution of lung ventilation of the four horizontal divisions at the following four time points: before anesthesia induction in a spontaneously breathing state, after anesthesia induction, before and after the LRM in a mechanically ventilated state at the end of surgery. We collected the EELI and regional distribution of lung ventilation for three respiratory cycles and averaged the value.

Arterial blood gas parameters, including arterial partial pressure of oxygen (PaO_2_) and ventilation-related parameters were collected at the following three time points: after anesthesia induction and before and after the LRM. Hemodynamic parameters were recorded as a video clip by an assistant researcher. A blinded independent researcher (EJ Oh) reviewed all video clips and recorded the lowest value of the following outcome variables during the LRM: systolic and mean arterial pressure, heart rate, and SpO_2_. According to the study protocol, if a patient showed hemodynamic instability after the first LRM (mean arterial pressure < 40 mmHg^17^ and/or heart rate < 40 bpm), the second LRM was not conducted. If hemodynamic instability, defined as a mean arterial pressure < 60 mmHg, heart rate < 50 bpm or > 120 bpm, and SpO2 < 90%, did not resolve in five minutes, it was corrected with intravenous crystalloid loading or vasoactive drugs.

### Endpoints and statistics

The primary aim of this study was to compare the maximal decrease in the systolic arterial pressure during the LRM between two body positions. The secondary aim was to compare changes in respiratory parameters, such as PaO_2_/FiO_2_, as an indicator of gas exchange and regional distribution of lung volumes as measured using EIT.

The desired sample size was calculated based on the results of a pilot study. In the pilot study, systolic arterial pressure decreased by 23.4 ± 14.8 mmHg during the LRM in group S, while this decrease was approximately 10 mmHg less in group L. Given this difference and assuming a power of 0.8, a type I error of 0.05, and a conservative dropout rate of 10%, at least 40 patients per group were required.

All data are expressed as number of patients (%), mean ± SD, or median (interquartile range). Normal distribution was evaluated by Kolmogorov–Smirnov test. Baseline characteristics were evaluated using the Student’s *t* test and chi-square test. Differences in parameters before and after the LRM were analysed using Student’s *t* tests. The incidence of hypotension and bradycardia were evaluated using the chi-square test. The difference in two time points, such as changes in regional distribution of lung ventilation in dorsal division before/after induction of anesthesia or LRM and changes in PaO_2_/FiO_2_ ratio between immediately after anesthesia induction and at the end of surgery were analysed using paired *t* tests. All analyses were performed using IBM SPSS software (IBM, Armonk, New York, USA). Statistical results of *P* < 0.05 were considered significant.

## Results

**Figure 2:**
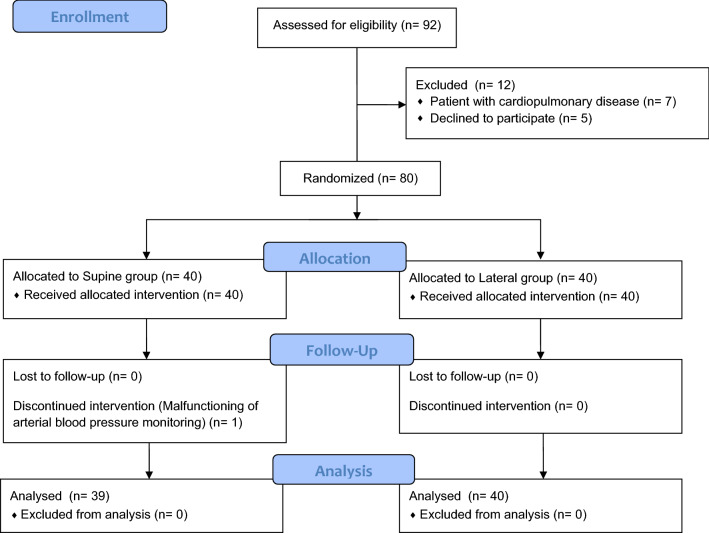
Consort flow diagram.

Of the 80 patients enrolled, one patient was excluded during the intervention because of a malfunction in the blood pressure monitoring system (Fig. 2). A total of 39 patients in group S and 40 patients in group L were assessed. There were no significant differences in patients or surgical characteristics between the two groups (Table 1). In both groups, the regional lung ventilation of the dorsal division was significantly reduced after anesthesia induction compared to before anesthesia (see Supplementary Fig. 1) and the PaO_2_/FiO_2_ ratio also significantly decreased at the end of surgery compared to immediately after anesthesia induction (P < 0.001, see Supplementary Fig. 2E).

**Table 1:** Baseline characteristics between the supine position group and the semi-lateral position group.

	Supine group (*n* = 39)	Semi-lateral group (*n* = 40)	*P *value
Age (year)	65.0 [59.0, 69.0]	63.5 [60.0, 67.8]	0.751
Height (cm)	166.2 [161.9, 168.5]	166.3 [162.6, 171.2]	0.578
Weight (kg)*	69.0 [62.8, 74.8]	71.0 [66.0, 76.0]	0.151
Body mass index (kg m^−2^)	25.2 [23.7, 26.3]	25.6 [24.2, 27.5]	0.187
Hypertension	14/39 (35.9)	15/40 (37.5)	0.883
Diabetes	3/39 (7.7)	6/40 (15.0)	0.307
**ASA class**			0.781
I/II/III	4/33/2	2/36/2	
**Smoking **s**tatus**			0.572
Never	16/39 (41.0)	21/40 (52.5)	
Former	18/39 (46.2)	14/40 (35.0)	
Current	5/39 (12.8)	5/40 (12.5)	
Ejection fraction in TTE	63.4 ± 4.5	63.7 ± 5.5	0.805
**Pre-operative respiratory related variables**			
Saturation in room air	98 [97, 100]	98 [96, 99]	0.777
* P*aO_2_/FiO_2_ ratio (mmHg)	543.4 [490.0, 624.9]	539.8 [470.2, 594.5]	0.579
* P*aO_2_ (mmHg)	320.6 [282.2, 360.2[	328.8 [268.9, 353.1]	0.787
**Intra-operative surgery related variables**			
Operation duration (min)	189.0 [161.0, 201.0]	190.0 [168.0, 204.5]	0.999
Anesthesia duration (min) (includes patient positioning and LRM duration)	231.1 ± 35	231.9 ± 30	0.919
Insufflation duration (min)	165.0 [141.0, 183.0]	164.0 [140.3, 181.5]	0.275
Fluid administration (mL)	800 [700, 1000]	975 [800, 1100]	0.085
Estimated blood loss (mL)	150 [100, 200]	175 [150, 200]	0.555
Intraabdominal pressure	14 [14]	14 [13, 14]	0.106
**Volume status before recruitment maneuver**			
Stroke volume variation	9 [7, 12]	9 [7, 12]	0.953
Pulse pressure variation	9 [6, 10]	9 [7,10]	0.697
**Hemodynamic parameters before recruitment maneuver**			
Stroke volume (L)	0.06 [0.06, 0.08]	0.07 [0.06, 0.08]	0.261
Cardiac index (L min^−1^ m^−2^)	2.4 [2.1, 2.7]	2.5 [2.1, 3.0)	0.238

Data are presented as the median [25th percentile, 75th percentile] or frequency (percent).

*ASA* American Society of Anesthesiologist, *TTE* transthoracic echocardiography.

### Hemodynamic changes during the LRM

Table 2 and Supplementary Fig. 2 shows the changes in hemodynamic parameters induced by the LRM. The systolic arterial pressure during the LRM decreased significantly in group S compared with group L ([−] 27.6 ± 14.6% vs. [−] 18.6 ± 9.9%, *P* = 0.001). The incidence of hypotension defined as systolic arterial pressure < 85 mmHg^18^ during LRM was significantly higher in group S compared with group L (76.9% vs. 55.0%, OR [95% CI] 2.72 [1.03–7.19], *P* = 0.04). The incidence of hypotension defined as mean arterial pressure < 60 mmHg^17^ during the LRM was also significantly higher in group S compared with group L (61.5% vs. 30.0%, OR [95% CI] 3.73 [1.47–9.51], *P* = 0.007). Change in heart rate was greater in group S than in group L (6.5 ± 6.4% vs. [−] 0.5 ± 11.3%, *P* = 0.001), and there was a higher incidence of bradycardia (< 60 bpm) in group S than in group L, (59.0% vs. 32.5%: 2.99 [1.19–7.49], *P* = 0.018). However, none of the patient showed desaturation (SpO_2_ below 90%). All cases of hypotension and bradycardia resolved within five minutes without rescue medication.

**Table 2:** Hemodynamic parameters difference during the sustained inflation lung recruitment maneuver.

	Supine group (*n* = 39)			Semi-lateral group (*n* = 40)			*P-*value
	Before LRM	During LRM	Difference (%) *	Before LRM^†^	During LRM^†^	Difference (%) *	
**Blood pressure**							
Systolic arterial pressure (mmHg)	102 [92, 113]	76 [65, 84]	(−) 27.6 ± 14.6	103 [94, 113]	83 [76, 96]	(−) 18.6 ± 9.9	0.001
Diastolic arterial pressure (mmHg)	58 [52, 64]	46 [42, 54]	(−) 9.8 ± 8.2	59 [53, 64]	52 [46, 62]	(−) 5.3 ± 9.3	0.023
Mean arterial pressure (mmHg)	76 [66, 85]	55 [49, 65]	(−) 18.1 ± 9.8	75 [71, 84]	63 [58, 72]	(−) 10.2 ± 8.1	< 0.001
**Heart rate**	61 [57, 69]	57 [52, 67]	6.5 ± 6.4	68 [59, 73]	65 [55, 75]	(−) 0.5 ± 11.3	0.001

Data are presented after normal distribution was assessed by Kolmogorov–Smirnov test. The systolic arterial pressure, diastolic arterial pressure, mean arterial pressure, heart rate before and during LRM are presented as median [25th percentile, 75th percentile], while the difference in values are presented as mean ± SD. *Difference is defined as the minimum values during the sustained inflation lung recruitment maneuver minus the value before lung recruitment maneuver and is expressed as a percent of the value before recruitment maneuver.

### Changes in PaO_2_/FiO_2_ ratio and lung volume distribution after the LRM

As shown in Table 3, the improvement in PaO_2_/FiO_2_ after the LRM was more prominent in group L than in group S (144.9 [87.3–228.2] vs. 69.9 [36.5–119.8], *P* < 0.001).

**Table 3:** Arterial blood gas analysis and respiratory system mechanic variables difference of before and after recruitment maneuver between groups.

	Supine group (*n* = 39)			Semi-lateral group (*n* = 40)			*P *value
	Before LRM	After LRM	Difference (%)	Before LRM	After LRM	Difference (%)	
**Arterial blood gas analysis**							
PaO_2_/FiO_2_ ratio	421.1 [385.1, 493.6]	488.2 [433.7, 548.9]	18.2 [8.4, 29.2] *	387.8 [334.6, 467.8]	560.5 [512.3, 596.8]	39.3 [20.2, 63.6] *	0.001
PaO_2_ (mmHg)	236.9 [192.8, 216.6]	306.1 [261.7, 323.3]	29.1 [17.2, 59.4] *	211.3 [177.8, 242.4]	323.2 [296.4, 346.3]	49.7 [34.5, 77.8] *	0.002
**Regional distribution of lung volume (%)**							
Ventral portion (V)	19.2 [12.6, 31.3]	21.2 [11.0, 33.0]	0.4 [(−)3.4, 6.1] ^†^	24.9 [13.4, 35.7]	23.8 [13.4, 37.1]	2.2 [(−)5.4, 7.6] ^†^	0.955
Mid-ventral portion (MV)	34.0 [28.3, 39.2]	34.0 [27.6, 39.4]	(−)0.3 [(−)3.7, 5.2] ^†^	36.0 [29.5, 40.3]	31.4 [23.3, 38.8]	(−)1.6 [11.1, 5.2] ^†^	0.148
Mid-dorsal portion (MD)	25.8 [17.3, 36.1]	24.5 [17.0, 33.0]	(−)1.3 [(−)6.1, 2.8] ^†^	23.4 [18.6, 31.8]	23.5 [16.6, 32.8]	(−)3.2 [(−)8.7, 9.3] ^†^	0.581
Dorsal portion (D)	15.2 [10.9, 20.4]	14.3 [10.5, 24.0]	(−)0.9 [(−)2.1, 4.3] ^†^	12.5 [8.1, 17.0]	18.0 [13.2, 22.7]	4.4 [(−)3.1, 10.2] ^†^	0.251

Data are presented as median [25th percentile, 75th percentile] after normal distribution was assessed by Kolmogorov–Smirnov test. *Difference is defined as the values after recruitment maneuver minus the values before recruitment maneuver and is expressed as a percent of the value before recruitment maneuver. ^†^, Difference is defined as the values after recruitment maneuver minus the values before recruitment maneuver. *P* values compare the difference of the value before and after RM between the two groups by student *t* test.

*LRM* lung recruitment maneuver, *PaO*_*2*_ partial pressure of oxygen, *FiO*_*2*_ fraction of inspired oxygen.

**Figure 3:**
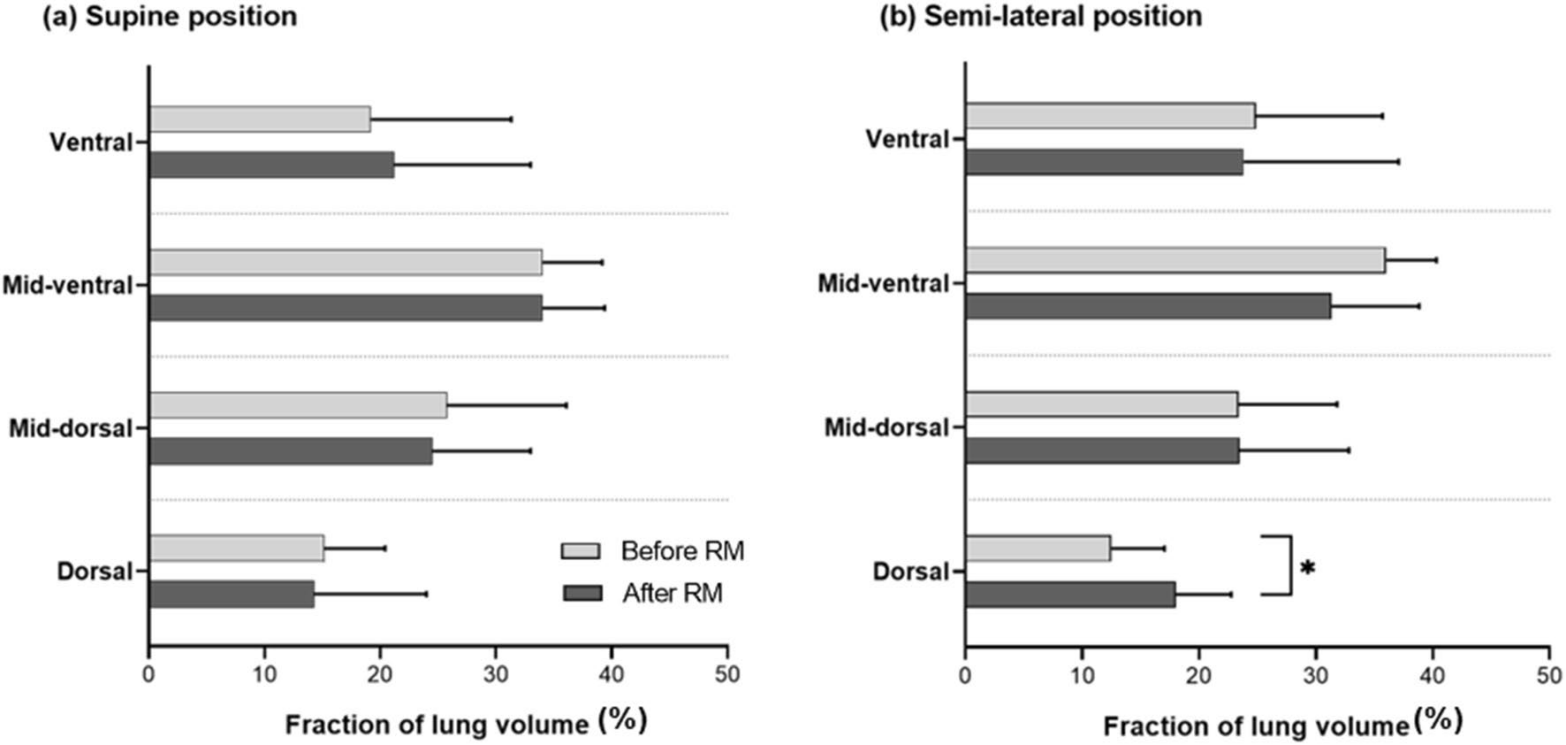
Changes in regional distribution of lung ventilation before and after the lung recruitment maneuver (LRM) in (**a**) supine position and (**b**) semi-lateral position. *P value < 0.05 by paired *t* test when comparing the regional distribution of lung ventilation in the dorsal division before and after the LRM. The bright gray bar indicates the fraction of lung ventilation before the LRM, while the dark gray bar indicates the fraction of lung ventilation after the LRM.

Regarding respiratory mechanics measured by EIT, the change in EELI was not statistically significant between the two groups (0.0 ([−]0.2–0.2) in group S vs. 0.1 ([−]0.5–0.7) in group L; *P* = 0.455). Interestingly, the regional distribution of lung ventilation changed differently depending on the patient’s body position during the LRM (Table 3; Supplementary Fig. 1). In both groups, lung volume in the dorsal division, the most dependent portion of the lung, decreased after anesthesia induction and remained the smallest region at the end of surgery (Supplementary Fig. 1; Supplementary Table 2). Accordingly, both groups did not show a difference in trend among four time points: before anesthesia, after anesthesia, at the end of surgery before LRM, and after LRM (*P* = 0.996 by repeated measure ANOVA among four time points). However, after performing the LRM, regional lung volume distribution differed between the two groups. In group S, lung volume in the ventral division (the most non-dependent portion) showed the greatest increase, while group L presented the largest increase in the dorsal division. As shown in Fig. 3 and supplementary Fig. 1, only the dorsal division in group L showed a statistically significant increase in regional lung volume distribution, from 12.5% of total lung volume before the LRM to 18.0% after the LRM (*P* = 0.024 by paired *t* test).

In terms of postoperative clinical outcomes, none of the study participants showed any pulmonary infection and no significant differences were found in the incidence of extension of oxygen therapy or desaturation in the post-anesthesia care unit (PACU) and length of hospital stay between the two groups (Supplementary Table 1).

## Discussion

This study was the first to observe the physiological benefits of a lung recruitment in the semi-lateral position in the operating room. The semi-lateral position induced less hypotension during the sustained LRM inflation and improved pulmonary gas exchange compared with the supine position, by effectively re-expanding the most dependent portion of the lung.

Transpulmonary pressure decreases along the vertical axis of the thorax due to the gravity-dependent increased weight of the lung by approximately [−]0.25 cmH_2_O for every 1 cm of ventral-to-dorsal thoracic diameter^19^. This increases the susceptibility of the dorsal lung division (the most dependent portion in a supine patient) to alveolar collapse during mechanical ventilation. However, the alveoli of the ventral division (the non-dependent portion) are usually kept open and aerated throughout surgery^20–22^. Regardless of patient position, the most dependent portion of the lung is always prone to atelectasis, resulting in impaired gas exchanges.

In this study, by changing patient position from supine to semi-lateral, the portion of the lung that was dependent during surgery became partially non-dependent. The overall area of the lung in contact with the surgical table was reduced and this may have improved the chest wall compliance during LRM. Moreover, increase in the vertical vector of the elevated lung may have resulted in an increase in transpulmonary pressure in this area and subsequently improved re-opening of the collapsed alveolar which was positioned in the dependent portion in supine. In other words, intrathoracic pressure can be transmitted more effectively to the collapsed alveoli in the semi-lateral position than in the supine position, while the reflected pressure to the heart is simultaneously reduced. Consequently, performing the LRM in the semi-lateral position was associated with less hemodynamic deterioration during lung inflation and better arterial oxygenation after the LRM compared with performing the LRM in the supine position.

Hypotension frequently occurs during sustained LRMs, and the occurrence of hemodynamic instability, even if temporary, halts the performance of the LRM and interferes with sufficient re-expansion of collapsed alveoli^23^. Because of the non-homogenous regional distribution of alveolar collapse, high transpulmonary pressure needed for “re-opening” the collapsed alveoli is a high “distending” pressure for normally aerated alveoli^24,25^. Thus, the alveoli in the non-dependent region may have higher risk for over-inflation during LRMs. In supine patients, this over-inflation has been reported to occur in the ventral division of lung^6^. This bilateral hyperinflation of the non-dependent portions of the lungs could potentially interfere with unrestricted venous return to the heart, which is located in the center of the iso-gravitational plane of the thorax (i.e., the non-dependent portion), resulting in a decrease in blood pressure^26,27^. In addition, the lung tissue stretching caused by hyperinflation could trigger the vagal reflex, causing bradycardia^28^.

In the present study, LRM in the semi-lateral position showed beneficial effects on hemodynamic stability, with minimal changes in blood pressure and heart rate. These hemodynamic benefits may be partly explained by improved chest wall compliance in the non-dependent thorax and less hyperinflation in the non-atelectatic alveolar at the given sustained inspiratory airway pressure compared with the supine position. As can be seen from the results observed by EIT, the increase of the lung volume distribution after the LRM was predominantly in the most ventral division in the supine group. However, in the semi-lateral group, the regional lung volume increased most after LRM in the dorsal division, where atelectasis frequently occurs during surgery. The result of more homogenous regional ventilation after LRM may have induced an improvement in oxygenation in the semi-lateral position.

Although the *P*aO_2_/FiO_2_ ratio increased after the LRM in both groups, this increase was significantly greater in the semi-lateral group reflecting a more effective re-aeration of the most collapsed alveoli in the dependent portion and consequently reducing intrapulmonary shunting. However, the EELI as measured by EIT, which represents the functional residual capacity (FRC), showed comparable increases in both groups. The EELI is known to measure the changes in electrical properties related to the ratio of total amount of air to fluid in a single scan near the diaphragm, while arterial oxygenation is associated with effective functional residual capacity at the location of gas exchange^9^. Therefore, the increase in lung volume measured by EIT could be the result of the same number of alveoli being more highly inflated rather than an increase in the number of aerated alveoli. This may have led to overestimation in the change in FRC^16^. This supports the importance of evaluating the effectiveness of the LRM considering both regional distribution of lung ventilation and arterial oxygenation.

Re-expanding collapsed alveoli is needed after prolonged laparoscopic surgery. As a part of the lung-protective strategy bundle, clinicians have been searching for optimal methods to effectively recruit the lung while minimizing alveolar over-distension and hemodynamic instability^29^. In this regard, our study has clinical implications in that this is the first to provide clinical evidence for the additional physiologic benefits of modified lung recruitment maneuver by simply changing the patient’s body posture to semi-lateral position. From the results of the current study, LRM in semi-lateral position was more effective to improve oxygenation and regional lung ventilation in the dependent portion of the lung without hemodynamic deterioration compared to supine position. However, we only included non-obese low-risk patients to facilitate the interpretation of the results and to standardize the research conditions other than the position of LRM. Therefore, the immediate physiologic benefits shown during and after LRM in our study did not lead to the clinically relevant outcome differences including postoperative pulmonary complication or the length of hospital stay. Nonetheless, we think this study is meaningful as an initial evidence of the LRM in semi-lateral position reporting feasibility and efficacy on clinical use. Although hemodynamic instability is a predictable adverse response during sustained lung inflation of the LRM, our study demonstrates evidence of relative hemodynamic stability during LRM in semi-lateral position with improvement in aeration. Based on the physiologic benefits, further studies among vulnerable patients with an increased risk of postoperative pulmonary complication, such as hemodynamically unstable patients who are difficult to apply high PEEP or obese patients with a high risk of atelectasis in the dorsal division of the lung, would be needed to confirm the clinical outcomes of LRM in semi-lateral position.

This study has some limitations. First, we included relatively healthy patients to observe physiological effects of LRMs in different body positions with minimized confounders. The physiological benefits of LRM in the semi-lateral position may be more apparent in vulnerable patients with cardiopulmonary disease. However, further studies are needed. Second, we did not measure the exact duration of hypotension and bradycardia. However, the duration of hemodynamic instability or its clinical effects could vary individually in more vulnerable patients and previous systemic review by Wesselink et al. showed that any exposures to MAPs below 55–50 mmHg reported increased risk of any end-organ injury^17^. Given the fact that hemodynamic instability is a predictable adverse response during sustained lung inflation of the LRM, we believe that modified measures to reduce it are clinically meaningful. Third, only male patients were included in our study. The combined risk of general anesthesia, intraoperative pneumoperitoneum and prolonged excessive Trendelenburg positioning in patients undergoing robotic assisted laparoscopic prostatectomy exacerbate atelectasis. We thought it would be appropriate to confirm the effectiveness of our modified LRM intervention in this high risk population. In addition, to facilitate the interpretation of the study results, we wanted to standardize the research conditions other than the LRM intervention. However, further studies including female patients or other types of surgery are needed to generalize our results. Fourth, in the present study, the LRM was conducted twice in the form of sustained inspiratory pressure. Although LRM using sustained inspiratory pressure is widely practiced form of LRM in the operating room^30^, research on various forms of LRM are needed to generalize the results. Fifth, lung compliance during LRM implementation was not observed in real time. If technically available, the pressure–volume curve could be checked, or LRM could be performed at lower airway pressure in the semi-lateral position through simultaneous scanning with lung ultrasound. Finally, because the flow parameters obtained from the EV1000™ system, such as stroke volume or cardiac index, are calculated every 20 s, the changes in hemodynamic data could not be displayed in real time during LRM. Further studies involving echocardiography or pulmonary arterial catheterization are needed for a clearer mechanistic interpretation of the lower hemodynamic deterioration seen during the LRM in the semi-lateral position compared to the supine position.

In this randomized-controlled study, the LRM in the semi-lateral position was effective in maintaining hemodynamic stability and increasing regional lung ventilation in the dependent portion of the lung, leading to an improvement in arterial oxygenation. Therefore, we suggest performing lung recruitments in the semi-lateral position to easily re-aerate the dependent portion of the lung and to prevent over-inflation of the non-dependent portion of the lung after laparoscopic surgery. Further studies are needed to investigate the effects of the semi-lateral position in different forms of recruitment maneuvers.

## Supplementary Information

Supplementary Information 1.

Supplementary Information 2.

Supplementary Information 3.

Supplementary Information 4.
