# Supplementary figures and images for: Physiological benefits of lung recruitment in the semi-lateral position after laparoscopic surgery: a randomized controlled study
Source: Sci Rep. 2022 Mar 10;12:3909. doi: 10.1038/s41598-022-04841-8 (PMC8913840; doi:10.1038/s41598-022-04841-8)

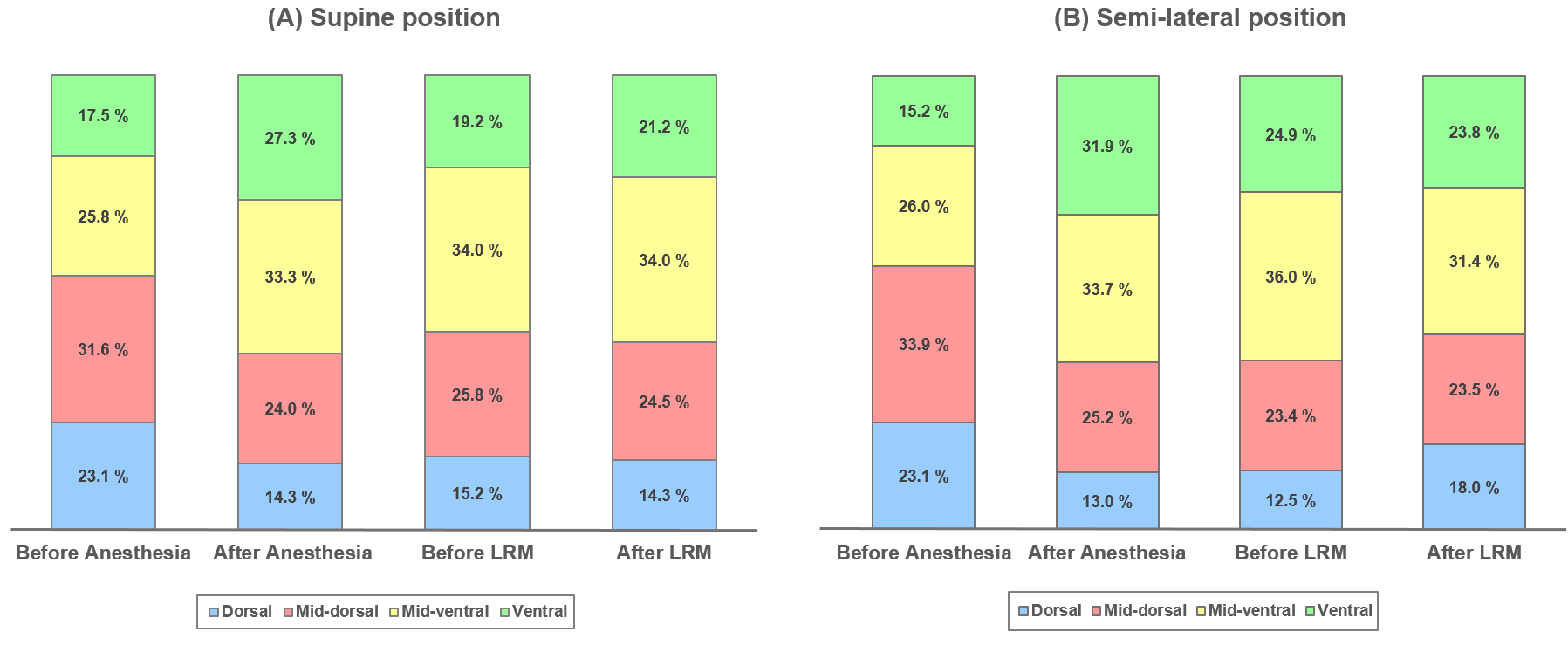

Supplement: Supplementary file 1 — Supplementary Information 1. [file 41598_2022_4841_MOESM1_ESM.tif]

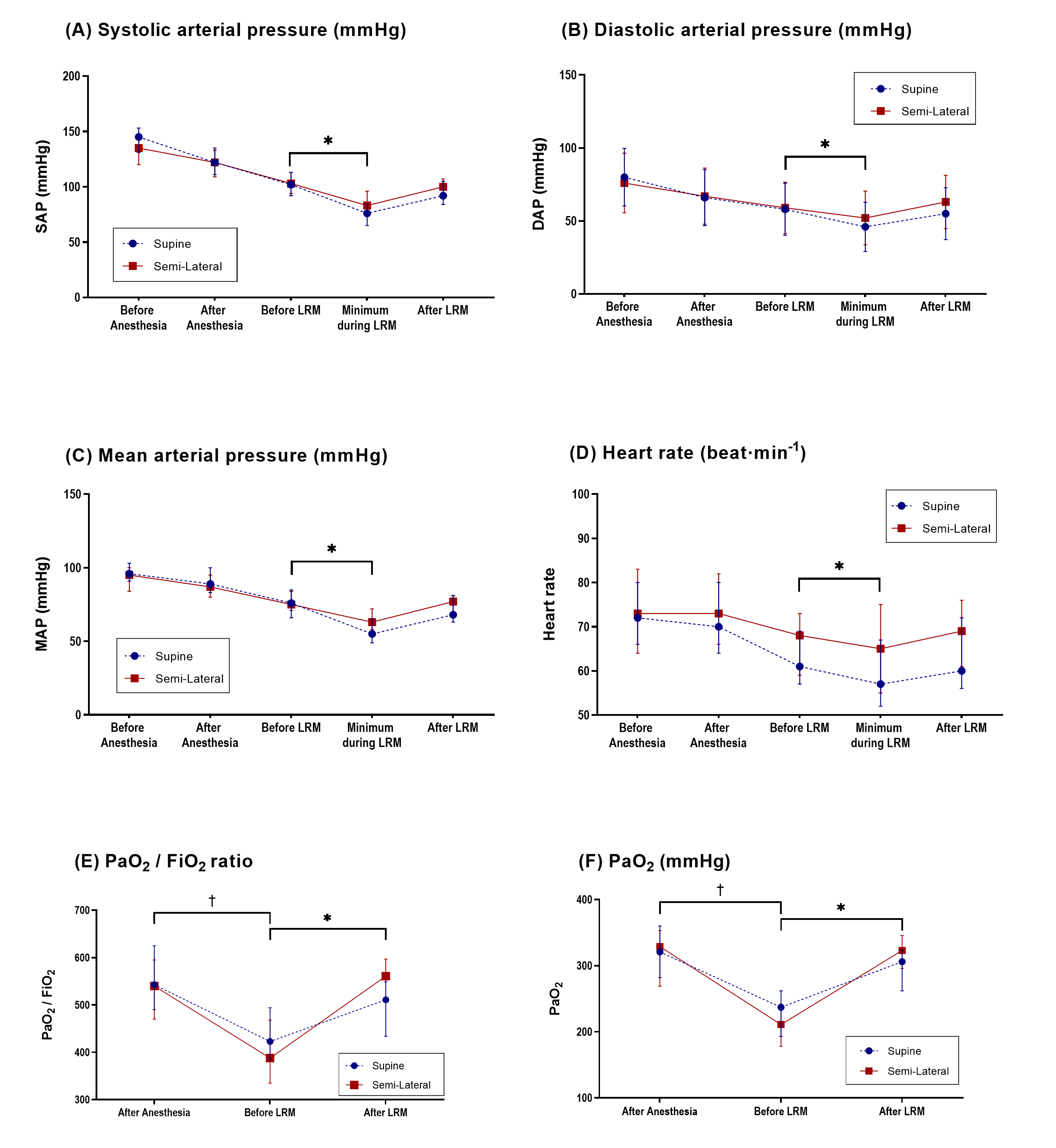

Supplement: Supplementary file 2 — Supplementary Information 2. [file 41598_2022_4841_MOESM2_ESM.tif]
