# Supplementary material for: Physiological benefits of lung recruitment in the semi-lateral position after laparoscopic surgery: a randomized controlled study
Source: Sci Rep. 2022 Mar 10;12:3909. doi: 10.1038/s41598-022-04841-8 (PMC8913840; doi:10.1038/s41598-022-04841-8)
Supplement: Supplementary file 3 — Supplementary Information 3. [file 41598_2022_4841_MOESM3_ESM.docx]

**Supplementary Figure 1.** Changes in regional distribution of lung ventilation of the four horizontal divisions at the following four time points in (A) supine position and (B) semi-lateral position: Before anesthesia, after anesthesia, before LRM, and after LRM.

**Supplementary Figure 2.** Changes in hemodynamic variables at the following four time points (Before anesthesia / after anesthesia / before LRM / Minimum during LRM / after LRM) and respiratory variables at the following three time points (After anesthesia / before LRM / after LRM) in supine position and semi-lateral position. (A) Systolic arterial pressure (mmHg), (B) Diastolic arterial pressure (mmHg), (C) Mean arterial pressure (mmHg), (D) Heart rate (beat·min^-1^), (E) PaO_2_ / FiO_2_ ratio, (F) PaO_2_ (mmHg). * indicates *P*-value <0.05 by paired *t*-test when the values before LRM and the minimum value during LRM are comprared for hemodynamic variables and the values before LRM and the values after LRM are comprared for respiratory variables. ^†^ indicates *P*-value <0.05 compared by paired *t*-test of the respriatory variables immediately after anestehsia induction and at the end of surgery before LRM.
