# Supplementary material for: Physiological benefits of lung recruitment in the semi-lateral position after laparoscopic surgery: a randomized controlled study
Source: Sci Rep. 2022 Mar 10;12:3909. doi: 10.1038/s41598-022-04841-8 (PMC8913840; doi:10.1038/s41598-022-04841-8)
Supplement: Supplementary file 4 — Supplementary Information 4. [file 41598_2022_4841_MOESM4_ESM.docx]

**SupplementaryTable 1.** Postoperative clinical outcomes between the supine position group and the semi-lateral position group.

|  | **Supine group (*n*=39)** | **Semi-lateral group (*n*=40)** | ***P-*value** |
| --- | --- | --- | --- |
| Extension of oxygen therapy in PACU | 12 / 39 (30.8%) | 10 / 40 (25.0%) | 0.622 |
| Oxygen desaturation in PACU | 2 /39 (5.1%) | 0 | 0.241 |
| Length of hospital stay (days) | 7 (7-7) | 7 (7-7) | 0.264 |

Data are presented as frequency (percent) or median (25^th^ percentile, 75^th^ percentile). PACU, post-anesthesia care unit. Oxygen desaturation in post-anesthesia care unit (PACU) was defined as patients who showed oxygen saturation below 90 percent in PACU and extension of oxygen therapy in PACU was defined as patients who required additional oxygen therapy after 30 minute of anesthesia recovery period in the PACU.

**Supplementary Table 2.** Hemodynamic variables, arterial blood gas analysis, and respiratory system mechanic variables difference between groups at four time points: before anesthesia, after anesthesia, before LRM and after LRM.

|  | **Supine group (*n*=39)** | | | |  |  | | **Semi-lateral group (*n*=40)** | | | | | | | |
| --- | --- | --- | --- | --- | --- | --- | --- | --- | --- | --- | --- | --- | --- | --- | --- |
|  | **Before anesthesia** | **After anesthesia** | **Before LRM** | **After LRM** | |  |  | | **Before anesthesia** | | **After anesthesia** | | **Before LRM** | | **After LRM** |
| **Hemodynamic variables** | | | | | | | | | | | | | | | |
| **Blood pressure** | | | | | | | | | | | | | | | |
| SAP (mmHg) | 145 [131, 153] | 122 [111, 135] | 102 [92, 113] | 92 [84, 105] | |  |  | | 135 [120, 147] | 122 [109, 133] | | 103 [94, 113] | | 100 [91, 107] | |
| DAP (mmHg) | 80 [74, 85] | 66 [61, 75] | 58 [52, 64] | 55 [49, 62] | |  |  | | 76 [69, 85] | 67 [61, 76] | | 59 [53, 64] | | 63 [54, 67] | |
| MAP (mmHg) | 96 [91, 103] | 89 [83, 100] | 76 [66, 85] | 68 [63, 81] | |  |  | | 95 [84, 100] | 87 [80, 95] | | 75 [71, 84] | | 77 [69, 81] | |
| **Heart rate** | 72 [66, 80] | 70 [64, 80] | 61 [57, 69] | 60 [56, 72] | |  |  | | 73 [64, 83] | 73 [66, 82] | | 68 [59, 73] | | 69 [61, 76] | |
| **Arterial blood gas analysis** | | | | | | | | | | | | | | | |
| PaO_2_/FiO_2_ ratio |  | 543 [490, 625] | 423 [385, 494] | 511 [434, 549] | |  |  | |  | 540 [470, 595] | | 388 [335, 468] | | 561 [512, 597] | |
| PaO_2_ (mmHg) |  | 321 [282, 360] | 237 [193, 262] | 306 [262, 323] | |  |  | |  | 329 [269, 353] | | 211 [178, 242] | | 323 [296, 346] | |
| **Regional distribution of lung volume (%)** | | | | | | | | | | | | | | | |
| Ventral portion (V) | 17.5 [6.0, 27.8] | 27.3 [10.1, 35.2] | 19.2 [12.6, 31.3] | 21.2 [11.0, 33.0] | |  |  | | 15.2 [5.6, 22.8] | 31.9 [16.4, 36.9] | | 24.9 [13.4, 35.7] | | 23.8 [13.4, 37.1] | |
| Mid-ventral portion (MV) | 25.8 [23.0, 29.4] | 33.3 [28.9, 38.7] | 34.0 [28.3, 39.2] | 34.0 [27.6, 39.4] | |  |  | | 26.0 [17.4, 33.3] | 33.7 [27.2, 39.0] | | 36.0 [29.5, 40.3] | | 31.4 [23.3, 38.8] | |
| Mid-dorsal portion (MD) | 31.6 [24.7, 42.2] | 24.0 [19.8, 33.0] | 25.8 [17.3, 36.1] | 24.5 [17.0, 33.0] | |  |  | | 33.9 [26.1, 42.4] | 25.2 [18.5, 30.6] | | 23.4 [18.6, 31.8] | | 23.5 [16.6, 32.8] | |
| Dorsal portion (D) | 23.1 [17.0, 28.4] | 14.3 [11.3, 17.0] | 15.2 [10.9, 20.4] | 14.3 [10.5, 24.0] | |  |  | | 23.1 [17.7, 29.4] | 13.0 [9.8, 16.8] | | 12.5 [8.1, 17.0] | | 18.0 [13.2, 22.7] | |

Data are presented as median [25th percentile, 75th percentile] after normal distribution was assessed by Kolmogorov-Smirnov test. *P*-values compare the difference of the value before and after RM between the two groups by student *t*-test. DAP, Diastolic arterial pressure; FiO_2,_ Fraction of inspired oxygen; LRM, Lung recruitment maneuver, MAP, mean arterial pressure; *P*aO_2_, Partial pressure of oxygen; SAP, systolic arterial pressure.
